# Characterisation of endogenous players in fibroblast growth factor‐regulated functions of hypothalamic tanycytes and energy‐balance nuclei

**DOI:** 10.1111/jne.12750

**Published:** 2019-07-08

**Authors:** Benediktas Kaminskas, Timothy Goodman, Andrew Hagan, Saverio Bellusci, David M. Ornitz, Mohammad K. Hajihosseini

**Affiliations:** ^1^ School of Biological Sciences University of East Anglia Norwich UK; ^2^ Department of Developmental Biology Washington University School of Medicine St Louis Missouri; ^3^ Cardio‐Pulmonary Institute Justus Liebig University Giessen Germany; ^4^ International Collaborative Centre on Growth Factor Research Life Science Institute Wenzhou Medical University Wenzhou Zhejiang Province China

**Keywords:** FGF functions in the hypothalamus, gene expression, tanycytes

## Abstract

The mammalian hypothalamus regulates key homeostatic and neuroendocrine functions ranging from circadian rhythm and energy balance to growth and reproductive cycles via the hypothalamic‐pituitary and hypothalamic‐thyroid axes. In addition to its neurones, tanycytes are taking centre stage in the short‐ and long‐term augmentation and integration of diverse hypothalamic functions, although the genetic regulators and mediators of their involvement are poorly understood. Exogenous interventions have implicated fibroblast growth factor (FGF) signalling, although the focal point of the action of FGF and any role for putative endogenous players also remains elusive. We carried out a comprehensive high‐resolution screen of FGF signalling pathway mediators and modifiers using a combination of in situ hybridisation, immunolabelling and transgenic reporter mice, aiming to map their spatial distribution in the adult hypothalamus. Our findings suggest that β‐tanycytes are the likely focal point of exogenous and endogenous action of FGF in the third ventricular wall, utilising FGF receptor (FGFR)1 and FGFR2 IIIc isoforms, but not FGFR3. Key IIIc‐activating endogenous ligands include FGF1, 2, 9 and 18, which are expressed by a subset of ependymal and parenchymal cells. In the parenchymal compartment, FGFR1‐3 show divergent patterns, with FGFR1 being predominant in neuronal nuclei and expression of FGFR3 being associated with glial cell function. Intracrine FGFs are also present, suggestive of multiple modes of FGF function. Our findings provide a testable framework for understanding the complex role of FGFs with respect to regulating the metabolic endocrine and neurogenic functions of hypothalamus in vivo.

## 1 INTRODUCTION

Growing evidence shows that, in addition to hypothalamic neurones, tanycytes act as sensors, mediators and effectors for critical processes that underpin the homeostatic and neuroendocrine functions of the hypothalamus; for example, sensing of metabolites and micronutrients and trafficking of peripheral and central signals such as leptin and gonadotrophin‐releasing hormone.^1^, ^2^ Moreover, a subset of tanycytes acts as bona fide neural stem/progenitor cells in the postnatal and adult hypothalamus, capable of generating new energy balance‐regulating neurones and glia.^3^, ^4^, ^5^ Tanycytes are residual radial glial‐like cells that occupy the floor and ventrolateral walls of the third ventricle (3V). They have been subdivided into four main subtypes: β2, β1 and α2, α1, according to a combination of projection trajectory, morphology, barrier properties, cilia arrangement and positioning within or outside marker domains within the 3V wall.^1^ Commonly, their apical surface is exposed to the cerebrospinal fluid, whereas their basal processes either contact the portal capillaries of the central (β2‐tanycytes) and lateral (β1‐tanycytes) median eminence, or terminate within the arcuate or ventromedial and dorsomedial nuclei (α‐tanycytes), where the neurones are crucial for maintaining energy homeostasis and related food‐seeking behaviour.

Fibroblast growth factors (FGFs) are emerging as important regulators of tanycyte biology and hypothalamic neuronal function. There are 22 mammalian FGFs in total, of which 18 function as paracrine, endocrine or autocrine molecules by activating one of four types of FGF receptors (FGFR1‐4) and the ensuing downstream intracellular signalling pathways: Ras‐Raf‐mitogen‐activated protein kinase (MAPK), phosphoinositide 3‐kinase‐Akt and phospholipase C γ.^6^, ^7^ The remaining FGFs (FGF11‐14) function intracellularly in a receptor‐independent manner.^8^ FGFR signalling can modulate diverse aspects of cell behaviour in a cell‐type specific manner. This is achieved partly via differential levels of signalling, established by intracellular negative feedback loop regulators such as Sproutys (Spry) and Map‐kinase phosphatases (Mkp).^9^ Tissue specificity of the action of FGF is governed by target cell expression of the so‐called IIIb or IIIc alternatively‐spliced receptor isoforms that engage a mutually‐exclusive set of FGFs.^10^ Moreover, co‐factors such as sulphated proteoglycans and β‐Klotho molecules not only facilitate focal FGF/FGFR signalling, but also determine the range of extracellular FGF diffusion. For example, peripherally generated FGF19 and FGF21 evade entrapment by heparan sulphate proteoglycans to act as circulating hormones in the metabolic control of energy homeostasis, with potential clinical applications in the management of type 2 diabetes.^11^, ^12^

Exogenous FGF2 is a potent mitogen for hypothalamic ependymal and neural cells in vitro and in vivo.^5^, ^13^ Levels of FGF1 show a dramatic postprandial rise in the cerebrospinal fluid,^14^, ^15^ whereas experimental elevation or attenuation of canonical FGF signalling, via peripheral or ic.v. application of FGF ligands, soluble FGFR fragments or neutralising antibodies, can induce hypo‐ and hyperphagia, respectively.^16^, ^17^, ^18^, ^19^ Exogenous FGF1 also accelerates glucose clearance and induces sustained remission of diabetes symptoms in a diabetic mouse model.^20^

A full understanding of how exogenous FGFs function and/or whether endogenous FGFs have similar or unique roles requires the identification and spatial distribution of the key players: ligands, receptors, receptor co‐factors and signalling modifiers. To address this need, we carried out a detailed survey of FGF signalling pathway components in the mediobasal hypothalamus, using reverse transcriptase‐polymerase chain reaction (RT‐PCR) screens, in conjunction with in situ hybridisation (ISH), immunolabelling and analysis of transgenic reporter mice. Our investigations reveal intricate and restricted domains of FGF and FGFR receptor expression amongst tanycytes, in addition to both distinct and overlapping patterns of receptor and ligand expression within the neighbouring energy balance regulating nuclei.

## 2 MATERIALS AND METHODS

### 2.1 Animals

Both male and female mice ranging in age from 30 to 80 days of age were used. All mice were bred and maintained on a mixed C57BL6/129Ola background, under a 12:12 hour light/dark cycle, in accordance with local and national regulations and licenses governing experimental work with animals. Tissues from *Fgf9‐*lacZ; *Fgf18*‐CreER and *Etv4‐*GFP transgenic reporter mice were kindly provided by Professors David Ornitz and Saverio Bellusci, as reported previously,^21^, ^22^ or recently generated strains. To detect *Fgf18*‐expressing cells in the brain, mice carrying *Fgf18*‐CreERT2::Rosa‐tdTomato‐dsred alleles were injected i.p. with tamoxifen (300 mg kg^‐1^ body weight) at postnatal day (P)50, P51 and P52, before tissue harvest at P53.

### 2.2 Tissue isolation and preparation

Mice were killed by CO_2_ asphyxiation and brains were then dissected out and fixed for 4‐16 hours overnight in 4% paraformaldehyde (PFA) (pH 7.0) at 4°C. For fresh tissue and mRNA isolation, mice were killed by cervical dislocation and microdissected hypothalami were flash frozen in cryotubes on dry ice and stored at −80°C. To generate cryostat sections, the brains were washed with diethyl pyrocarbonate (DEPC)‐treated PBS (DEPC‐PBS) and cryo‐protected in 30% w/v sucrose/DEPC‐PBS solution for 48 hours at 4°C. Thereafter, they were placed in cryomoulds filled with optimal cutting temperature (OCT) compound, allowed to set on dry ice, and stored at −80°C. Coronal sections, 20 μm thick, containing the central part of median eminence (bregma −1.45 to −1.94) were then generated using a freezing microtome (MicroM HM560; Thermo Fisher Scientific, Waltham, MA, USA) and thaw‐mounted onto previously baked (180°C, overnight) Superfrost (Thermo Fisher Scientific) slides, and stored at −80°C until use.

To generate vibratome sections, PFA‐fixed brains were first dehydrated in ascending concentrations (30%, 50%, 70%, 90%; 1 hour per dilution) of ethanol, before being stored at 4°C in absolute ethanol. Just before use, brains were rehydrated stepwise back to PBS and embedded in 3% w/v agar overnight. Coronal sections, 60 μm thick, containing median eminence (bregma −1.46 to −2.18) were then generated using a vibrating microtome (Leica Microsystems, Wetzlar, Germany) and stored in PBS until use.

### 2.3 RNA isolation

Microdissected hypothalami were homogenised by trituration in 1 mL of Trizol (Invitrogen, Carlsbad, CA, USA) before addition of 200 μL of chloroform. Following centrifugation (15 minutes at 12 000 *g*), RNA was precipitated from the aqueous layer by addition of 500 μL of isopropanol. Samples were briefly vortexed, allowed to stand for 10 minutes at room temperature and then centrifuged (15 minutes at 12 000 *g*). The resulting supernatant was carefully removed before adding 1 mL of 75% ethanol diluted in molecular grade water (Sigma, St Louis, MO, USA) and further centrifugation (12 minutes at 5200 *g*). The RNA pellet was then briefly air dried, re‐suspended in molecular grade water and incubated at 55°C for 10 minutes. RNA concentration and quality was determined using a spectrophotometer (Nanodrop ND‐1000; Thermo Fisher Scientific) and samples were stored at −80°C until use.

### 2.4 Primer design and RT‐PCR

Gene specific primer pairs (see Supporting information, Table S1) for use in RT‐PCR reactions were designed using NCBI primer‐BLAST ( https://www.ncbi.nlm.nih.gov/tools/primer-blast), aiming for regions of least sequence conservation between related family members,^23^ and amplicons that are not only small, but are also comparable in size across the related family members (see Supporting information, Table S1). To rapidly generate gene‐specific templates for in vitro transcription of antisense cRNA probes (see below), some reverse primers were additionally tagged at their 5′ end with a T7 polymerase‐encoding promoter sequence (TAATACGACTCACTATAGGG).^23^ RT‐PCR reactions were carried out in a thermocycler (MJ Research, Waltham, MA, USA) using *illustra Ready‐To‐Go™* RT‐PCR beads (GE Healthcare Life Sciences, Marlborough, MA, USA) and 1 μg of hypothalamic RNA per reaction. For first strand synthesis, Oligo (dT)_12‐18_ primers (Invitrogen) and the following cycles were used: 15 minutes at 42°C; 5 minutes at 95°C; and 15 minutes at 6°C. After the addition of 1.5 pmol of forward and reverse gene‐specific primers, PCR was carried out using: 30 seconds at 95°C, then 34 cycles of 30 seconds at 60°C; 1 minute at 68°C; followed by 15 minutes at 6°C. The products were resolved alongside 1Kb DNA ladder on 1% w/v agarose gel and visualised using ethidium bromide. RT‐PCR reactions with β‐actin primers served as a positive control.

### 2.5 Riboprobe synthesis and In situ hybridisation (ISH)

Digoxigenin‐labelled antisense RNA probes were generated using T7 RNA polymerase enzyme (Thermo Fisher Scientific) and either T7‐tagged RT‐PCR amplicons (above) or linearised plasmids encoding gene‐specific cDNAs (see Supporting information, Table S2), as templates. Riboprobes were purified using Chroma Spin columns (Clontech, Mountain View, CA, USA) and stored at −20°C until use.

ISH reactions were performed after optimisation of previously reported protocols,^24^ as detailed below. Briefly, cryosections were allowed to equilibrate at room temperature for 1 hour before a 5 minutes fixation in 4% PFA, followed by three 5 minutes washes with DEPC‐PBS. Sections were then treated for 50 minutes with 1 μg mL^‐1^ proteinase K in a buffer of 50 mmol L^‐1^ (pH 8.0) Tris‐HCl and 5 mmol L^‐1^ ethylenediaminetetraacetic acid and then post‐fixed with 4% PFA for 5 minutes. After three 5‐minutes washes with DEPC‐PBS, sections were incubated for 10 minutes in acetylation solution (1.4% triethanolamine, 0.2% HCl, 0.25% acetic anhydride). Prehybridisation was carried out for 4 hours in hybridisation buffer (50% formamide, 5 × salt sodium citrate buffer (SSC), pH 7.0, 5 × Denhardt's solution, 0.25 mg mL^‐1^ salmon sperm DNA, 0.5 mg mL^‐1^ yeast tRNA). Hybridisation was carried out at between 68 and 72°C in a hybridisation oven for 16‐18 hours, using 500 ng mL^‐1^ riboprobes diluted in hybridisation buffer. Post hybridisation washes were: 5 minutes with 5 × SSC at room temperature, followed by three 30 minutes washes with 0.2 × SSC at hybridisation temperature and a wash with 0.2 × SSC at room temperature. Sections were then washed with Tris‐HCl/saline buffer (100 mmol L^‐1^ Tris‐HCl, pH 7.5, 150 mmol L^‐1^ NaCl) at room temperature and blocked in Tris‐HCl/saline buffer with 10% heat‐inactivated normal goat serum (hiNGS) before an overnight incubation at 4°C with alkaline phosphatase‐conjugated anti‐digoxigenin conjugated antibodies (see Supporting information, Table S2) diluted in Tris‐HCl/saline buffer, containing 3% hiNGS. Excess primary antibody was removed by six 15‐minutes washes in Tris‐HCl/saline buffer. Colour reaction was carried out in NTM buffer (0.1 mol L^‐1^ Tris‐HCl, pH 9.5, 0.1 mol L^‐1^ NaCl, 50 mmol L^‐1^ MgCl_2_) containing 2 mmol L^‐1^ levamisole, 375 μg mL^‐1^ 4‐nitro blue tetrazolium chloride and 187.5 μg mL^‐1^ 5‐bromo‐4‐chloro‐3‐indolylphosphate, and allowed to progress at 4°C until a signal became visible. The reactions were stopped by three 5‐minute washes with PBS, followed by a 1‐hour wash in 95% ethanol. Sections were then rehydrated for 15 minutes in ddH_2_O before being coverslipped in 50%/PBS glycerol.

Negative controls included the omission of antisense and/or use of sense cRNA probes (*Fgf9, Fgf20, Klb, Sef, Spry1, Spry2*). Positive controls included probes for Neuropeptide Y (Npy), an anorexigenic neurotransmitter expressed by some arcuate neurones, which invariably yielded a very strong and specific signal.

### 2.6 Immunohistochemistry, immmunofluorescence labelling and X‐gal staining

Non‐specific binding sites were blocked by a 2‐hour incubation in a solution of 20% hiNGS/1% Triton X‐100 (TX)/PBS, before application of primary antibodies (see Supporting information, Table S2) overnight at 4°C in 0.2% hiNGS/0.1% TX/PBS. Excess primary antibodies were removed by five 1‐hour washes at room temperature with a 0.2% hiNGS/0.1% TX solution and this was followed by overnight incubation with the relevant secondary fluorophore conjugated antibodies (see Supporting information, Table S2) diluted in 0.2% hiNGS/0.5% NP‐40/in PBS at 4°C. The next day, sections were washed six times (30 minutes per wash) before counterstaining with nuclear DNA marker, Hoechst, and subsequent mounting in Vectashield (Vector Laboratories, Burlingame, CA, USA).

For combined ISH and immunohistochemistry, after completion of the ISH protocol, sections were incubated for 1 hour in blocking solution (10% hiNGS/0.3% TX/PBS) followed by overnight incubation at 4°C with mouse anti‐glial fibrillary acidic protein (GFAP), rabbit anti‐GFAP or mouse anti‐S100β antibodies (see Supporting information, Table S2) diluted in 0.2% hiNGS/0.1% TX/PBS. After three 5‐minute PBS washes, the 3,3’‐diaminobenzidine (DAB) staining reaction was performed using a DAB peroxidase substrate kit (Vector Laboratories) in accordance with the manufacturer's instructions. After five 1‐minute washes with ddH_2_O, sections were preserved by coverslipping with 50% glycerol/PBS solution.

To detect the product of the lacZ reporter gene, β‐galactosidase, brains of *Fgf10*‐lacZ reporter mice were preserved in Mirsky's fixative (National Diagnostics, Atlanta, GA, USA) overnight at 4ºC, washed three times with PBT (PBS with 0.1% Tween‐20) before being incubated with the X‐gal substrate solution (2 mmol L^‐1^ MgCl_2_, 5 mmol L^‐1^ K_4_Fe(CN)_6_‧3H_2_O, 0.02% v/v NP‐40, 0.1% v/v X‐gal; diluted in PBS) at 37ºC for 30 minutes. Samples were then post fixed with 4% PFA and then dehydrated into absolute ethanol for subsequent use and sectioning (see above).

### 2.7 Image capture and analysis

All images were captured using a Axioplan 2 microscope and axiovision, version 4.8 (Carl Zeiss, Oberkochen, Germany). High magnification of phophorylated extracellular signal‐regulated kinase (pERK) immunostaining was imaged and analysed by 3D reconstruction of 1‐μm optical sections (z stacks), captured using an Apotome attachment. Images were post‐processed (adjustment of brightness, contrast, levels and unsharp mask) using fiji ( https://fiji.sc), axiovision and photoshop (Adobe Systems Inc., San Jose, CA, USA) software packages.

## 3 RESULTS

### 3.1 Global analysis: detection of distinct FGFs and signalling modulators and predominance of FGFR IIIc isoforms

To determine which components of the FGF signalling system need to be mapped spatially in the hypothalamus, we first undertook a gross RT‐PCR screen of mRNA expression derived from 30‐45‐day‐old (P30‐P45) animals. Industrially pre‐aliquoted substrates (illustra Ready‐To‐Go beads; GE Healthcare Life Sciences) were used to amplify comparable amplicon sizes. We opted for an RNA‐based and gene‐reporter analysis (here and below) because antibodies to FGFs often detect multiple related family members and, in the case of FGFRs, they fail to discriminate between isoforms in immunolabelling studies. Animals older than P30 were used because the full spectrum of tanycytes subtypes, as well as the connectivity of hypothalamic neurones, is fully established by this age.^25^

**Figure 1:**
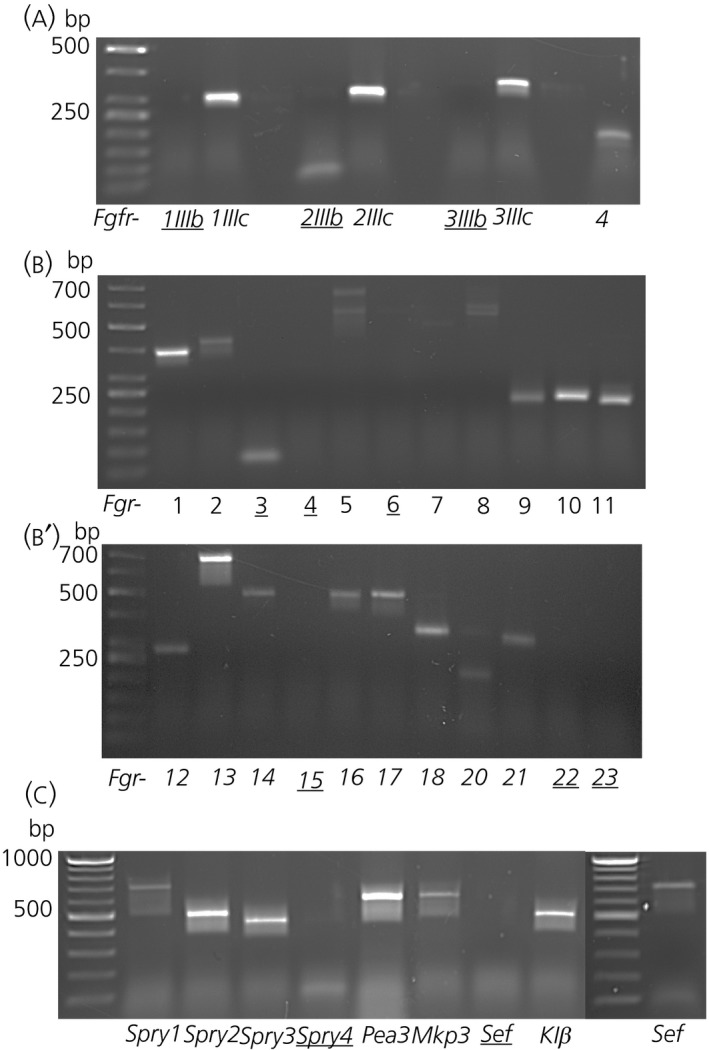
Gross reverse transcriptase‐polymerase chain reaction (RT‐PCR) survey of fibroblast growth factor (FGF) ligands, receptors, signalling modulators and mediators in the postnatal hypothalamus. RT‐PCR products for FGF receptors 1 to 4 (A); *Fgf1*‐*23* (B, B′); and FGF signalling modulators and mediators (C). Underlined numbers and letters denote a lack of detection of the relevant genes. Faint bands for *Fgfr2 IIIb* isoform and *Fgf3* are non‐specific products, whereas all other RT‐PCR products match the expected amplicon size, as predicted by use of primers listed in the Supporting information (Table S1). Multiple *Fgf5* and *Fgf8* products possibly represent isoforms of these genes

FGFR1‐3 exist as alternatively‐spliced isoforms termed “IIIb” and “IIIc”, depending on alternate usage of exons that encode the amino half of the third extracellular immunoglobulin‐like domain of receptors, whereas FGFR4 possesses only the IIIc form. Use of isoform‐specific primers showed that FGFR1‐3 are the predominantly expressed receptors, specifically, their IIIc isoforms (Figure 1A), suggesting that IIIc‐activating FGF ligands are the likely drivers of canonical FGFR signalling in the hypothalamus.

Use of primer pairs for all 22 mouse *Fgfs* (see Supporting information, Table S1) detected products of the expected size for *Fgf1, 2, 5, 7, 8, 9, 10, 11, 12, 13, 14, 16, 17, 18, 20* and *21* (Figure 1B,B′). Multiple bands were detected for *Fgf5* and *Fgf8*, possibly representing their closely‐related isoforms.^26^ Co‐receptor β‐Klotho, which is required for the action of FGF19 and FGF21, as well as FGFR signalling modulators *Spry1*,* Spry2*,* Spry3* and *Mkp3* but not *Sef* or *Spry4*, were also detectable, with *Spry2* and *Spry3* showing stronger expression (Figure 1C). *Pea3* (also known as *Etv4*), a transcriptional target of FGF signalling, was also present.

This broad screen reveals that various components of the FGF signalling pathway are in place to mediate exogenous and to potentially augment endogenous FGF signalling in the hypothalamus.

### 3.2 Predominance of FGFR1 and FGFR2, in the β‐tanycyte domain

The gross RT‐PCR screen (above) was performed using bulk tissue that contains multiple cell compartments: the median eminence (ME), the ependymal cell wall and the hypothalamic parenchyma. To delineate specific expression of FGF signalling apparatus in tanycytes and the flanking mediobasal nuclei (arcuate [ARC], ventromedial [VMN], dorsomedial [DMN] and lateral hypothalamic area [LHA]), at high resolution, we performed mRNA ISH on coronal sections containing the central part of the ME (bregma −1.45 to −1.94) from P60‐P80 mice. Because the expression of astroglial marker, GFAP, is normally prominent in the α‐tanycyte domain and dorsal ependymal cells, and absent from the β‐tanycyte domain^1^, ^4^ (see Supporting information, Figure S1), the ISH analysis was combined with mouse anti‐GFAP immunolabelling to investigate the selective expression of candidate genes within α‐ vs β‐tanycyte subtypes.

**Figure 2:**
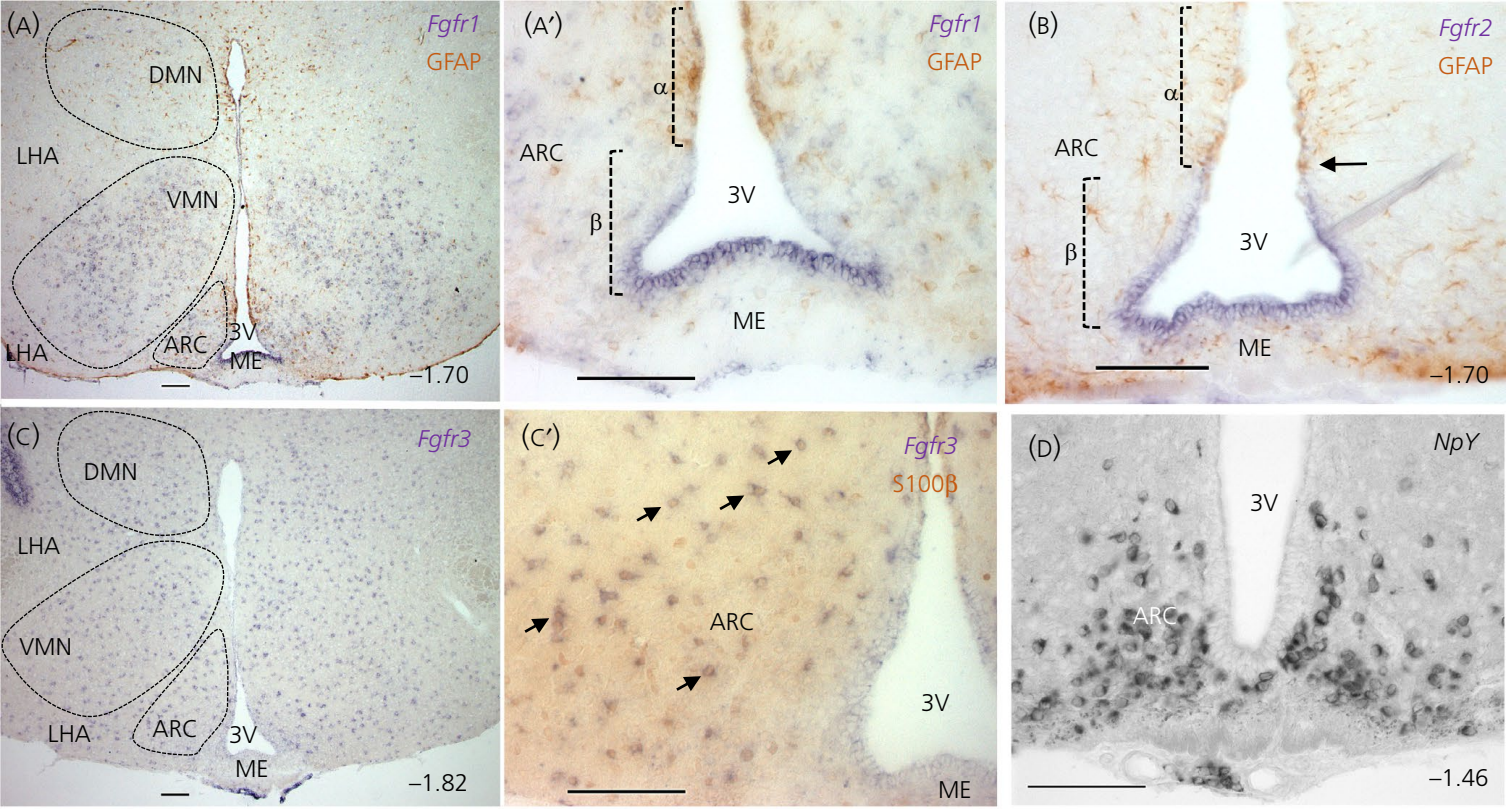
Domain‐restricted expression of fibroblast growth factor (FGF) receptors within the walls of the third ventricle (3V) and the parenchyma of the mediobasal hypothalamus. Representative images of in situ hybridisation experiments using *Fgfr1* (A, A′), *Fgfr2* (B) and *Fgfr3* (C, C′) cRNA probes combined with immunohistochemistry for glial fibrillary acidic protein (GFAP) (A, A′) or S100β (C′), and a positive control probe, Neuropeptide Y (Npy) (D). *Fgfr1* and *Fgfr2* staining in the third ventricle wall is mostly restricted to β‐tanycytes (A, A′, B). *Fgfr2* expression extends into the transition zone (arrow in B) bordering β‐ (GFAP^−^/S100β^−^) and α‐ (GFAP^+^/S100β^+^) tanycytes. *Fgfr1* is also expressed in hypothalamic nuclei, outlined by dashed lines (A). (C) Distribution of *Fgfr3‐*expressing cells in hypothalamic parenchyma, with most co‐expressing glial cell marker S100β (C′, several examples arrowed). (D) *Neuropeptide Y (NpY)* in the rostral part of the arcuate nucleus. Approximate bregma co‐ordinates in bottom right corners. Scale bars = 100 μm. ARC, arcuate nucleus; DMN, dorsomedial nucleus; LHA, lateral hypothalamic area; ME, median eminence; VMN, ventromedial nucleus

We found that *Fgfr1* and *Fgfr2* are expressed predominantly in the GFAP‐ve β‐tanycyte domain (Figure 2A,A′,B). Interestingly, the strongest *Fgfr1* signal was observed in the floor of the 3V, which harbours the β2 subtype (Figure 2A′), whereas *Fgfr2* was equally strong in both β2‐ and β1‐tanycyte domains, as well as the transition zone between β1‐ and α‐tanycytes (Figure 2B). *Fgfr1* expression was also detected in cells of the ARC, DMN and LHA, with strongest expression in the VMN (Figure 2A). By contrast, *Fgfr2* riboprobes only weakly stained cells in the ARC, VMN, DMN and LHA, as well as subependymal cells of the ME (Figure 2B; data not shown).

Interestingly, *Fgfr3* expression was absent from the ependymal cell wall altogether, instead being abundant in cells scattered uniformly throughout the hypothalamic parenchyma, reminiscent of glial cell distribution (Figure 2C), as well as spare cells in the ME. We combined *Fgfr3* in situ with immunolabelling for glial/progenitor cell marker, S100β, only to find prevalent co‐expression of these markers, confirming the glial identity of most *Fgfr3*‐expressing cells (Figure 2C,C′). Glial expression of *Fgfr3* is precedented in the embryonic mouse spinal cord, where *Fgfr3* delineates most GFAP+ astrocytes.^27^ Use of Digoxygenin‐labelled *Fgfr4* cRNA probes did not yield a stain in our ISH reactions, consistent with a recent quantitative RT‐PCR profiling of NPY‐GFP+ sorted arcuate cells.^28^

### 3.3 Expression of FGF signalling modulators, mediators and targets overlaps with FGFRs

**Figure 3:**
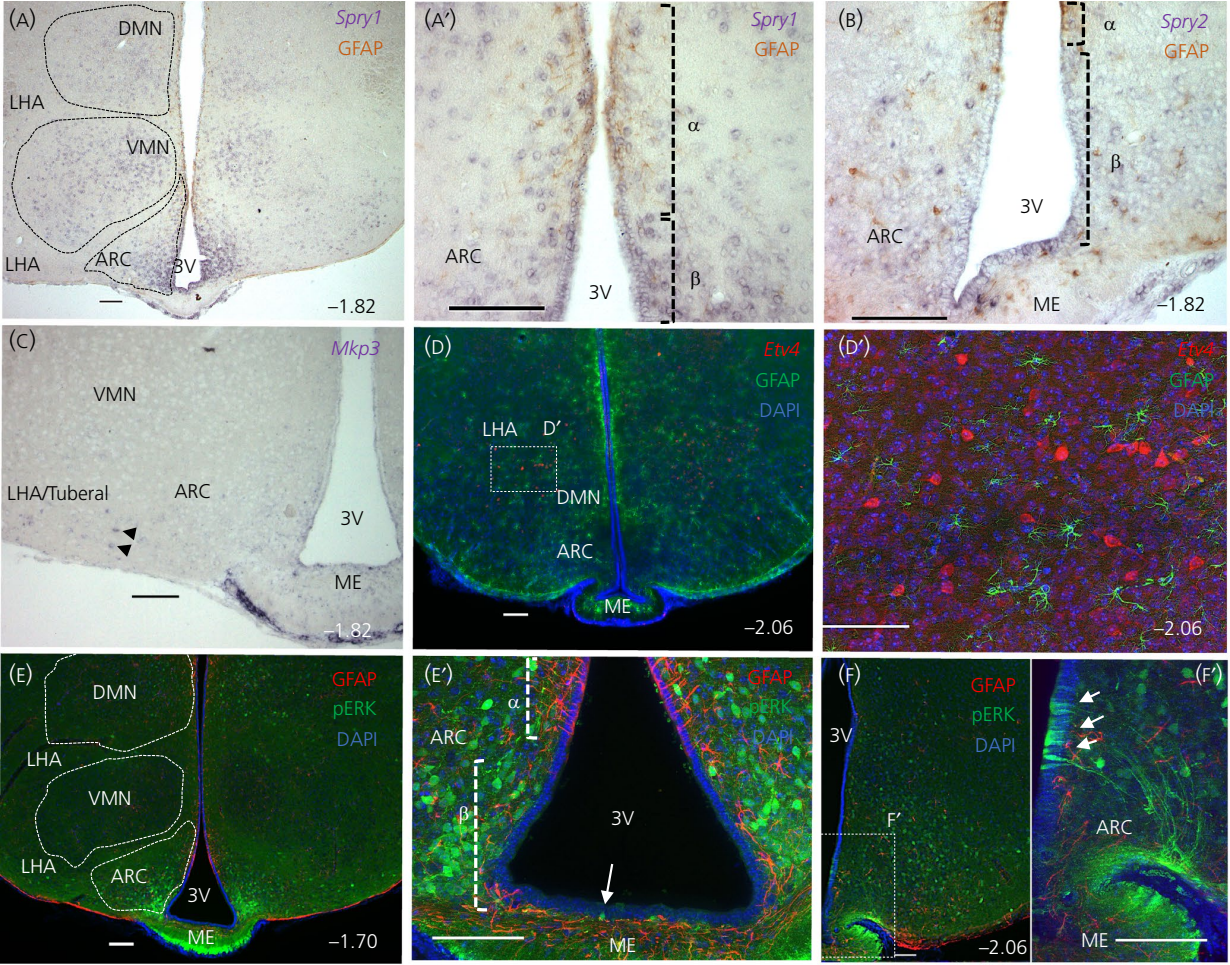
Focal expression of fibroblast growth factor (FGF) signalling modifiers and mediators. Representative images of *Spry1* (A, A′), *Spry2* (B), *Mkp3* (C) in situ hybridisation reactions and anti‐GFP immunolabelling of *Etv4‐*GFP brain sections (D, D′), alone or in combination with glial fibrillary acidic protein (GFAP) *(*A, A′, B, C, D, E, E′) or phophorylated extracellular signal‐regulated kinase (pERK) (E, E′, F) immunolabelling. *Spry1* is found in β‐ and α‐tanycyte domains, as well as hypothalamic parenchyma (A, A′); *Spry2* is observed in β‐tanycytes and sporadic cells in hypothalamic parenchyma (B). *Mkp3* (C, black arrowheads) and *Etv4* (D, D′) are both found only in an area corresponding to the tuberal nucleus. pERK staining is strong in dorsomedial (DMN) and arcuate (ARC) nuclei. Although sparse in ependymal layer of 3V anteriorly (E, E′, white arrow), the foci of the pERK signal is evident caudally in β‐tanycytes (F, F′, bregma −2.06, arrows). Bregma co‐ordinates as indicated in right corners; Scale bars = 100 μm. DAPI, 4’,6‐diamidino‐2‐phenylindole; LHA, lateral hypothalamic area; ME, median eminence; VMN, ventromedial nucleus

A key feature of FGF signalling is the transcriptional activation of its own downstream negative regulators to fine tune intracellular levels of signalling.^9^ Exploiting this phenomenon, we investigated the domains of *Spry* family and *Mkp3* expression to determine exactly where endogenous FGFR signalling may be occurring in the mediobasal hypothalamus. ISH with *Spry1* riboprobes (Figure 3A,A′) yielded a strong signal in β‐tanycytes and cells of the ARC, although expression in VMN, DMN and LHA was also evident. Co‐labelling with GFAP antibodies revealed a weak expression in the α‐tanycyte domain (Figure 3A′). By contrast, use of *Spry2* riboprobes (Figure 3B) showed a more restricted pattern, composed of a weak signal in β‐tanycytes, and scattered expression in the ARC, LHA and subependymal ME. *Spry3* ISH reactions produced a weak background signal. *Mkp3* signal was restricted to scant cells in the LHA/tuberal nucleus (Figure 3C).

Another transcriptional target of FGF signalling is the transcription factor *Etv4 (Pea3)* [16‐19]. To detect the domain of its expression, we double‐labelled hypothalamic sections of an *Etv4*‐GFP reporter mouse with anti‐GFP as well as anti‐GFAP antibodies and observed a GFP signal in cells of caudal VMN and LHA (caudal to bregma −1.94) but not in tanycytes or GFAP+ parenchymal astrocytes (Figure 3D,D′).

The MAPK/ERK signalling pathway is a major transducer of FGFR signalling and is inhibited by Sprouty proteins.^9^ To detect focal activation of this pathway, we immunolabelled for pERK and found a positive signal in parenchymal cells (Figure 3E), particularly in the ARC, DMN and LHA but not the VMN. With respect to the ependymal compartment, however, we noted a rostrocaudal difference in pERK stain, with scarce signal in α‐ and β‐tanycytes at bregma −1.70 (Figure 3E′), contrasting with distinct clusters of pERK‐positive β1‐tanycytes in more caudal sections, at bregma −2.06 and beyond, until the 3V recedes (Figure 3F,F′).

### 3.4 FGF ligands are expressed in tanycytes in two distinct patterns

The presence of FGFRs (IIIc isoforms) and FGF signalling modulators in the hypothalamus is suggestive of a necessity for cognate ligand(s). To delineate the cohort of putative ligands involved, we performed ISH using *Fgf*‐specific cRNA probes and used transgenic reporter mice. Four FGF candidates and two main domains of expression emerged from this screen.

**Figure 4:**
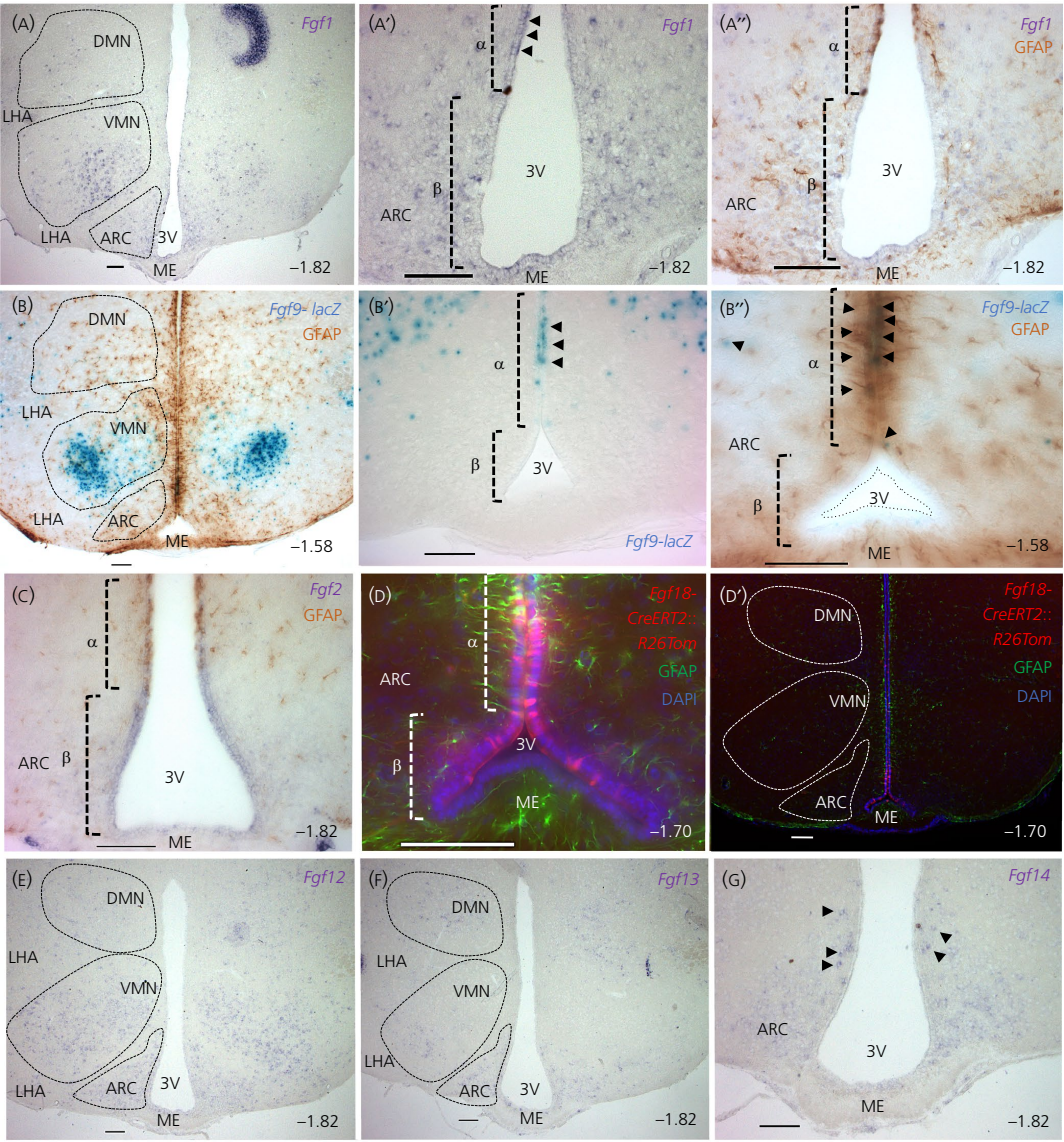
Distribution of paracrine‐ and intracrine‐acting fibroblast growth factors (FGFs) in the hypothalamus. Representative images showing in situ hybridisation reactions to detect *Fgf1* (A‐A″)*, Fgf2* (C) and *Fgf12‐14* (E‐G); X‐gal stain to detect *Fgf9* in *Fgf9*‐lacZ reporter mice (B‐B″); and tomato‐dsred immunolabelling to detect *Fgf18* in tamoxifen‐treated *Fgf18*‐CreERT2::R26‐Tomato‐dsred mice (D, D′). Additional immunolabelling or immunohistochemistry for glial fibrillary acidic protein (GFAP) in a subset of reactions to reveal the approximate domains of β‐ and α‐tanycytes (A″, B, B″, C, D, D′). Note the similarity in the domains of *Fgf1* and *Fgf9* expression (A‐B″), with a restriction to α‐tanycyte domain in the ependymal wall (black arrows in A′ and B″), prominence in the VMN, contrasting with the absence of *Fgf2* and *Fgf18* in the parenchymal compartment (C‐D′) and their prevalence in both α‐ and β1‐tanycyte domains, with a weak salt and pepper *Fgf2* expression in the β2 domain. Intracellular *Fgf12*‐*14* also predominate in the parenchymal compartment (E‐G), with *Fgf12* showing a broader domain of expression (E). Bregma co‐ordinates as indicated to the right; Scale bars = 100 μm. 3V, third ventricle; ARC, arcuate nucleus; DAPI, 4’,6‐diamidino‐2‐phenylindole; DMN, dorsomedial nucleus; LHA, lateral hypothalamic area; ME, median eminence; VMN, ventromedial nucleus

The expression domain of *Fgf1* and *Fgf9* overlapped in the 3V wall with a restriction to the α‐tanycyte domain (Figure 4A′‐A″,B′‐B′). Interestingly, ventricular expression of *Fgf9*, as judged by X‐gal staining of *Fgf9*‐lacZ brain, was also confined to rostral sections and absent from caudal bregma co‐ordinate −1.82 onwards. Parenchymal staining for these FGFs (Figure 4A,B; see also Supporting information, Figure S2) was most intense in the VMN, with sporadic staining of cells in the ARC, DMN and LHA. By contrast, expression of *Fgf2* and *Fgf18* encompassed both β1‐ and α‐tanycytes (Figure 4C,D; see also Supporting information, Figure S2). *Fgf2* expression in the α‐tanycyte domain tapered off sharply dorsally and so is therefore likely confined to the more ventral α2‐tanycyte subtype and, ventrally, a weak patchy pattern of expression was seen in β2‐tanycytes. *Fgf18* expression was determined by activation of the tdTomato‐dsred marker upon tamoxifen treatment of *Fgf18*‐CreERT2::Rosa‐tdTomato‐dsred mice (3‐day tamoxifen pulse; 1‐day chase; n = 2). A tight cluster of tdTomato‐expressing (Tom+) cells was observed spanning β1 and ventral α‐tanycyte domains, in addition to scarce Tom+ cells in dorsal parts of the 3V epithelium, well beyond tanycyte domain (not shown). It is noteworthy that the tracing paradigm used in the present study to report *Fgf18* expression would also report descendants of *Fgf18*‐expressing cells. Therefore, scarce Tom+ cells in the dorsal 3V epithelium may reflect dispersion of *Fgf18*‐expressing cells away from a focal source/site of *Fgf18* expression, which is an interpretation that is consistent with the *Fgf18* ISH data reported by Robins et al.^5^

*Fgf5* and *Fgf17* cRNA probes yielded a clear signal in the hippocampus (see Supporting information, Figure S3) indicative of successful ISH reactions, although none in the hypothalamus. Probes for *Fgf7, 8* and *20* gave a diffuse non‐specific signal, not too dissimilar from sense probes, and probes for *Fgf16* and *Fgf21* did not produce any staining (not shown).

### 3.5 Restriction of intracellular FGFs to the parenchymal cell compartment

ISH with cRNA probes for intracellular FGFs (11 to 14) revealed a predominant expression in the hypothalamic parenchyma and subependymal cells in the ME but a notable absence from tanycytes themselves. *Fgf12* expression showed the broadest pattern, being present in the DMN, ARC and LHA, most strongly in the VMN (Figure 4E). *Fgf13* was restricted to sparse cells in the DMN and ARC and rarely in cells of LHA and ventrolateral VMN (Figure 4F; data not shown). *Fgf14* was expressed by only a few cells in the most dorsomedial part of the ARC (Figure 4G).

## 4 DISCUSSION

**Figure 5:**
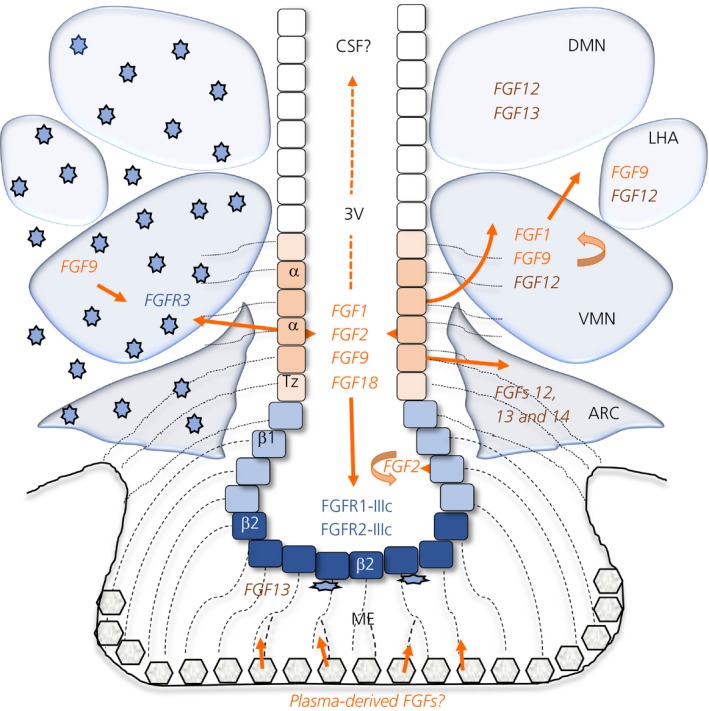
Schematic of key fibroblast growth factor (FGF) signalling partners and their putative modes of function in the mediobasal hypothalamus. Schematic coronal cross‐section of hypothalamus from approximate bregma −1.82. Square cobbles: cells lining of the third ventricle (3V), showing the approximate positioning of tanycyte subtypes, including a transition zone (Tz) between α and β subtypes, and their radial processes (thin dashed lines). Grey pentagonal boxes: capillary plexus in the outer zone of the median eminence (ME), contacted by β‐tanycytes. Blue shading denotes the domains of FGF receptor expression within β‐tanycytes, hypothalamic nuclei, scant subependymal cells in the ME and in parenchymal glial cells (star‐shaped), as putative targets of the action of FGF. Navy blue denotes overlap between *Fgfr1* and *Fgfr2* expression in the floor of the 3V. Orange and brown represent the domains of canonical (FGF1, 2, 9 and 18) and non‐canonical (FGF11‐14) expression. Paracrine (orange lines) and autocrine (curved orange lines) effects may involve FGF1, 2, 9 and 18, released by tanycytes into the 3V lumen or supplied to parenchymal cells by α‐tanycyte terminals, or produced by parenchymal cells themselves. Plasma‐derived FGFs could potentially activate FGF receptors via distal β‐tanycyte processes. FGFs released into lumen of 3V may circulate and have distant functions beyond the mediobasal hypothalamus (dashed arrows). FGF12, 13 and 14 would play non‐receptor mediated intracrine functions within the cells of the parenchymal nuclei, most prominently in ventromedial and arcuate, and some subependymal median eminence cells. ARC, arcuate nucleus; CSF, cerebrospinal fluid; DMN, dorsomedial nucleus; LHA, lateral hypothalamic area; ME, median eminence; VMN, ventromedial nucleus

Extrinsic modulation of FGF signalling in rodents can elicit dramatic changes in metabolism, body weight and plasma glucose clearance, with the hypothalamus being envisaged as an anchor for these effects. The therapeutic use of FGFs requires a better understanding of the foci and mechanisms by which FGFs operate, and whether endogenous hypothalamic FGF signalling normally has a role to play in these processes. On face value, our results, summarised in Table 1 and Figure 5, identify β‐tanycytes as the primary target and FGFR1 and FGFR2 IIIc isoforms as the main mediators of exogenous or endogenous canonical FGF signalling within the ependymal wall of the 3V. Moreover FGFR1 and FGFR3 appear as key players in hypothalamic parenchyma but with divergent roles. Our study predicts that FGF1, 2, 9 and 18, sourced mainly from tanycytes and cells of the VMN, are the key FGF ligands in any endogenous effects on ependymal and parenchymal cells. Endogenous FGF signalling is indicated by intricate expression of FGF pathway modulator and mediators, such as Sproutys and pERK. Collectively, our data form an integrative working model (Figure 5) for testing the regulatory role of FGFs in critical hypothalamic functions.

**Table 1:** Distribution of key FGF signalling players within different cellular compartments of the mediobasal hypothalamus

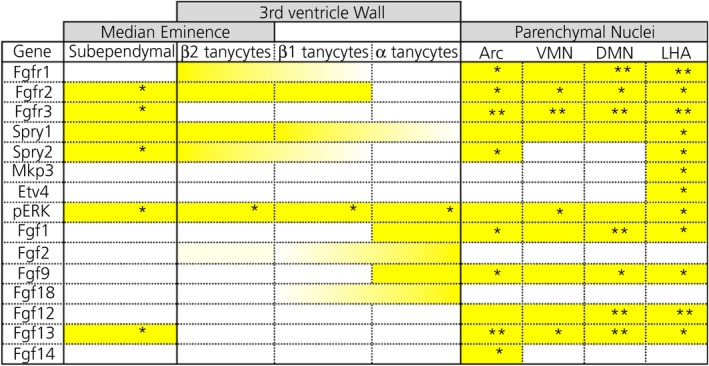


Note

Tapered colouring denotes a gradient of intensity of staining (darker ends, higher levels). A single star (*), denotes scattered cells expressing the gene and double stars (**) denote higher levels of expression or tighter clusters of gene‐expressing cells.

### 4.1 Comparison with previous profiling studies of the FGF signalling pathway in the hypothalamus

Several surverys and atlases^29^, ^30^, ^31^, ^32^ have documented the presence of FGFs/FGFRs in the hypothalamus, although most are either of low resolution, or of embryonic stages prior to the formation of tanycytes, or are presented in the saggital plane, which does not show the full cohort of 3V ependymal cells and tanycyte subtypes. Expression of *Fgfr1* and *Fgfr2 by* tanycytes had been noted in coronal sections (Allen Brain Atlas http://portal.brain-map.org/), although their precise cellular domain of expression had not been assessed. Recent studies show that the 3V epithelium is highly compartmentalised,^1^, ^33^, ^34^ and so we took advantage of general GFAP exclusion from the β‐tanycyte domain to show that *Fgfr1* and *Fgfr2* are mostly restricted to this tanycyte subtype. Additionally, co‐labelling with S100β enabled us to show that hypothalamic *Fgfr3* is mostly associated with glial cells. Noteworthy here is the growing importance of hypothalamic glial cells in the modulation of energy balance‐regulating neural circuits, via neuroinflammatory processes or otherwise.^35^ Much less was known about the domains of FGF signalling modulators and, in the present study, we highlight Spry1 and Spry2 as putative negative regulators of FGF signalling in tanycytes and hypothalamic neurones, although SPRYs can also operate downstream of other signalling pathways. With respect to FGF ligands, we cannot exclude the possibility that other endogenous FGFs, such as FGF7, 8, 16, 20 and 21, which are detected by RT‐PCR but not in ISH reactions, are also involved in hypothalamic functions. Exogenously‐applied FGF8 and FGF17 can certainly improve glucose homoestasis via neurones of the arcuate nucleus.^28^ Variations across species and/or differential levels of *Fgf* expression are also possible because *Fgf5* was detectable by ISH in the rat hypothalamus^36^ but not in the present study, despite the success of our probes in detecting *Fgf5* in the hippocampus (see Supporting information, Figure S3) in a similar pattern to that reported previously.^37^ The presence of FGF pathway genes that we have characterised at celllular resolution is supported by a recent drop‐seq gene profiling of cells in the median eminence and the ARC.^34^ However, it is possible that, with the use of more sensitive methods, such as isotopic ISH or quantitative PCR on cell sorted subpopulations, new and additional members may be identified.

### 4.2 Potential modes and significance of FGF/FGFR function in tanycytes and parenchymal nuclei

Because canonical FGF signalling requires the activation of FGFRs, specifically their IIIc isoforms in the hypothalamus (Figure 1A), FGFRs become the focus of debate when considering FGF function. Activation of FGFRs can yield a multitude of effects, typically involving cell proliferation, differentiation, migration or cytoskeletal remodelling.^7^ We showed that FGFR1 and FGFR2 are restricted to β‐tanycytes, where they could regulate cell proliferation and differentation of these newly‐identified stem/progenitor cells.^38^ Indeed, some studies have envisaged changes in postnatal hypothalamic neurogliogenesis as a contributory mechanism to body weight change and insulin‐independent glucose lowering effects of exogenous FGFs.^11^, ^39^ Equally, these effects may involve short‐ or long‐term alterations in barrier properties, nutrient sensing, and cargoing or trafficking of metabolic signals and hormones by β‐tanycytes.^2^, ^38^, ^40^ These cells are certainly enriched in the endocytotic pathway molecules, such as caveolins,^41^ which have been shown to control FGF2/FGFR1 signalling in other cell types and settings.^42^ Within the 3V epithelium, β‐tanycytes also uniquely possess primary cilia,^33^ as well as receptors for VEGFR3,^43^ and other key regulators of energy homeostasis such as ciliary neurotrophic factor^44^ and leptin.^38^ This suggests that β‐tanycytes may also be responsive to Hedgehog signalling and/or act as a hub of cell signalling, or that their biology relies on synergy between multiple signalling pathways. Therefore, fine dissection of other signalling pathways in tanycytes is warranted.

β2‐tanycytes are radial glial‐like cells whose processes span the width of the median eminence. Therefore, it is possible that FGFR1 and FGFR2 operate not only on their apical surfaces, exposed to ventricular‐derived FGFs, but also on their basal surfaces, in close proximity to the capillary plexus of the outer ME, where capillary fenestrations would readily allow exposure to circulating FGFs. Hints for the heterogeneity and paracine response of β‐tanycytes to endogenous FGFs comes from caudal expression of pERK in β1‐tanycytes, complemented by rostral expression of *Fgf9* in α‐tanycytes (Figure 4B). The rostrocaudal complimentarity of ligands and receptor is intriguing but highly reminiscent of developmental settings, where mesenchymal and epithelial cells express mutually exclusive cohorts of FGF ligands and receptors to ensure paracrine and uni‐directional FGF signalling.^9^ In the hypothalamus, FGFs that are secreted into the 3V space may well be distributed by beating cilia of 3V ependymal cells^45^ to activate distant receptors. This would be akin to critical asymmetrical distribution of FGF8 and Nodal by beating cilia during establishment of left‐right asymmetry in early vertebrate embryos.^46^ Similarly, strong expression of pERK in the ARC (Figure 3E) and *Etv4‐GFP* and *Mkp3* in the LHA is, complemented by strong expression of *Fgf9* in the neighbouring VMN (Figure 4B). ERK activation could also reflect receptor activation by FGF19 and FGF21 and leptin molecules that have crossed the blood‐brain barrier. Others studies have suggested that the suprachiasmatic nucleus is the foci of the action of FGF19 and FGF21 because their co‐receptor β‐Klotho is predominantly expressed by this nucleus.^32^

The functioning modes of FGF10 and FGF12‐14 are more engimatic. Within the ependymall wall, *Fgf10* expression is also restricted to β‐tanycytes,^4^, ^47^ although the target receptor, FGFR2 ‐IIIb^10^ is absent from the hypothalamus (Figure 1A). Similarly, FGF12‐14 do not normally engage FGFRs but are expressed by cells of the hypothalamic parenchyma. Increasing evidence, however, shows that FGFs may also function non‐canonically; for example, by trafficking to the nucleus/nucleolus, either alone, or in complex with FGFRs, to induce nuclear cell signalling or to carry out gene‐regulating functions via chromatin and RNA‐binding properties.^48^, ^49^, ^50^ FGF13 and FGF14, respectively, regulate the stabilisation of microtubules^51^ and proper functioning of voltage‐gated sodium and potassium ion channels^52^, ^53^ and so these intracellular FGFs could regulate axonal integrity and transport as well conductivity within neurones of the arcuate (Figure 4F,G) and other hypothalamic nuclei.

### 4.3 Lessons from human mutations and transgenic mouse models of the FGF signalling pathway

Is the prediction of FGFR1‐3 IIIc and FGF1, 2, 9 and 18 as key endogenous canonical FGF signalling partners in mouse hypothalamus, borne out by naturally‐occuring human mutations or experimental transgenic alleles? With minor exceptions (e.g. murine *Fgf15* vs human *Fgf19*), both the human and mouse FGF signalling apparatus is highly conserved in expression domain and function. Moreover, tanycytes have also been identified in the human hypothalamus^54^ and human and rodents broadly share common mechanisms for the regulation of energy balance. A spectrum of dominant acting mutations in human FGFR1 and FGFR2 causes rare congenital skeletal syndromes, such as Apert and Pfeiffer.^6^ However, no association between these syndromes and predisposition or resistance to diabetes and obesity has been reported. By contrast, a significant number of achondroplasia patients that carry activating FGFR3 mutations also manifest atypical obesity.^55^ Difficulties that prevent drawing firm conclusions from human patients include: the germline nature of these mutations, which would additionally impact peripheral organs or indeed multiple brain regions; their rareness, which may preclude statistically valid associations with metabolic/neuroendocrine defects or diet; and their varied molecular mode of function, with some mutant receptors operating in a FGF ligand‐independent manner.

With respect to engineered mouse models, germline deletion of FGFR1 and FGFR2, or FGF9 or FGF18^56^ causes prenatal or early postnatal lethality, whereas the loss of FGFR3 induces abnormal bone growth. FGF1‐ and FGF2‐deficient mice show no overt abnormalities, even as compound mutants. After challenge with a high‐fat diet, however, these mice show hyperglycaemia and insulin resistance, likely as a result of the critical regulation of adipose tissue by FGF1.^57^ Mice carrying a gain‐of‐*Fgfr1* function mutation appear normal,^58^ although those with a gain‐of *Fgfr2* mutation are hypoglycaemic and show growth retardation and early death.^59^ Fortunately, a plethora of conditional loss and gain of function alleles for these and other FGFs and FGFRs have been developed to circumvent embryonic/neonatal lethality. Their use in cell‐type specific and stage‐dependent gene targeting, as exemplified by recent work^28^, ^60^ will prove valuable for unravelling the exact role(s) of endogenous FGF signalling, alone or in combination with other signalling pathways, in tanycyte biology and the regulation of hypothalamic neuronal functions.

## Data Availability

The data that support the findings of the present study are available from the corresponding author upon reasonable request.
