# Supplementary figures and images for: Characterisation of endogenous players in fibroblast growth factor‐regulated functions of hypothalamic tanycytes and energy‐balance nuclei
Source: J Neuroendocrinol. 2019 Jul 8;31(8):e12750. doi: 10.1111/jne.12750 (PMC6772024; doi:10.1111/jne.12750)

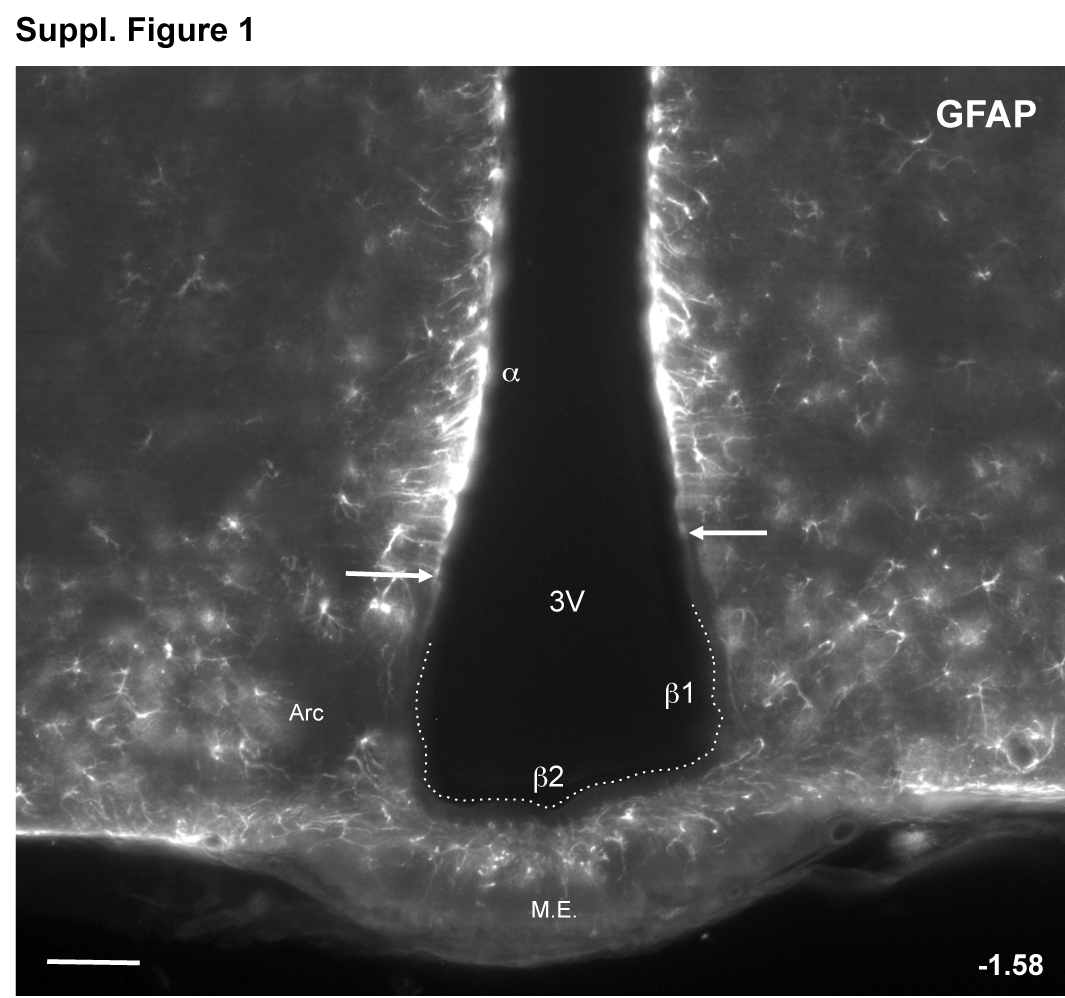

Supplement: Supplementary file 1 [file JNE-31-na-s001.tif]

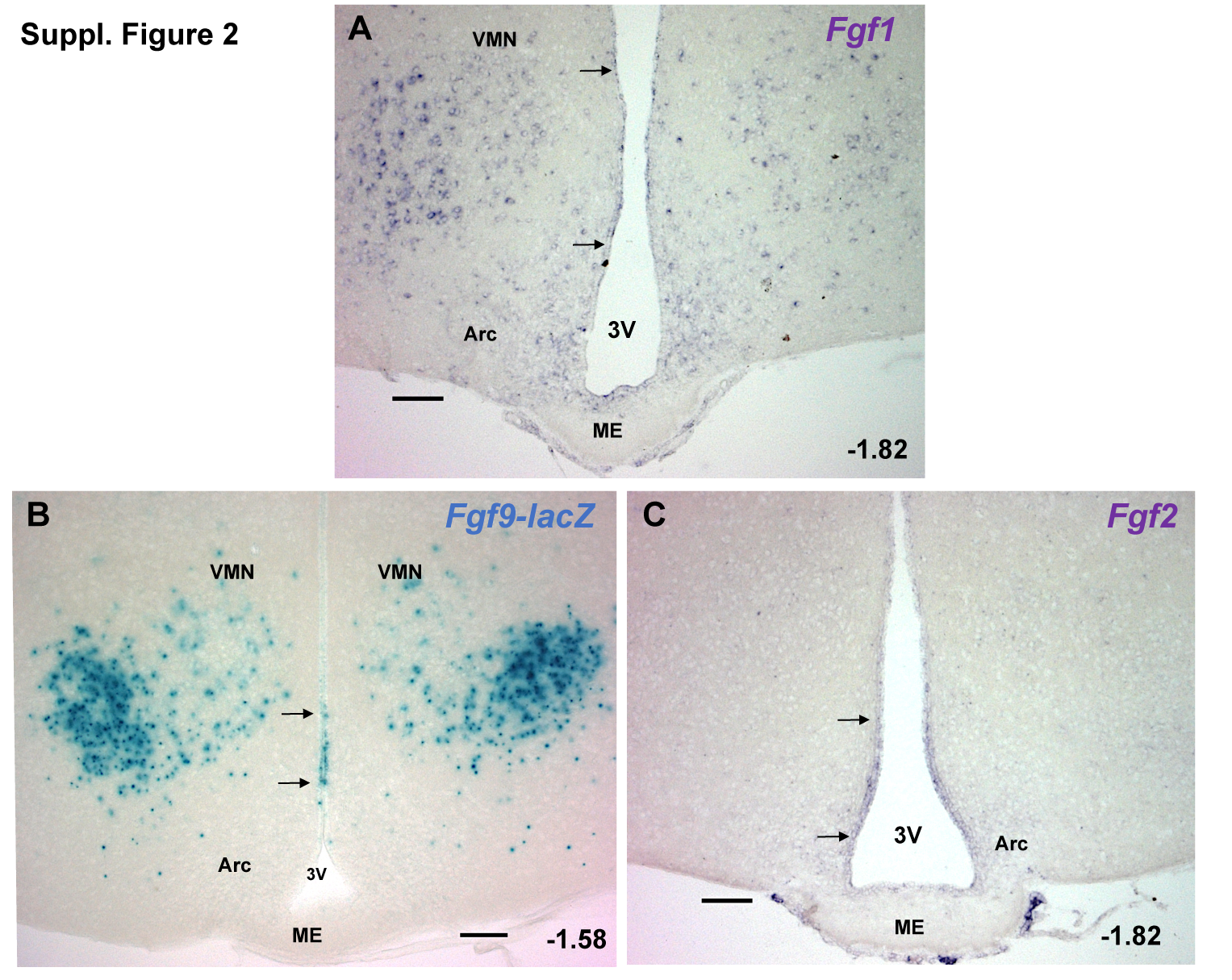

Supplement: Supplementary file 2 [file JNE-31-na-s002.tif]

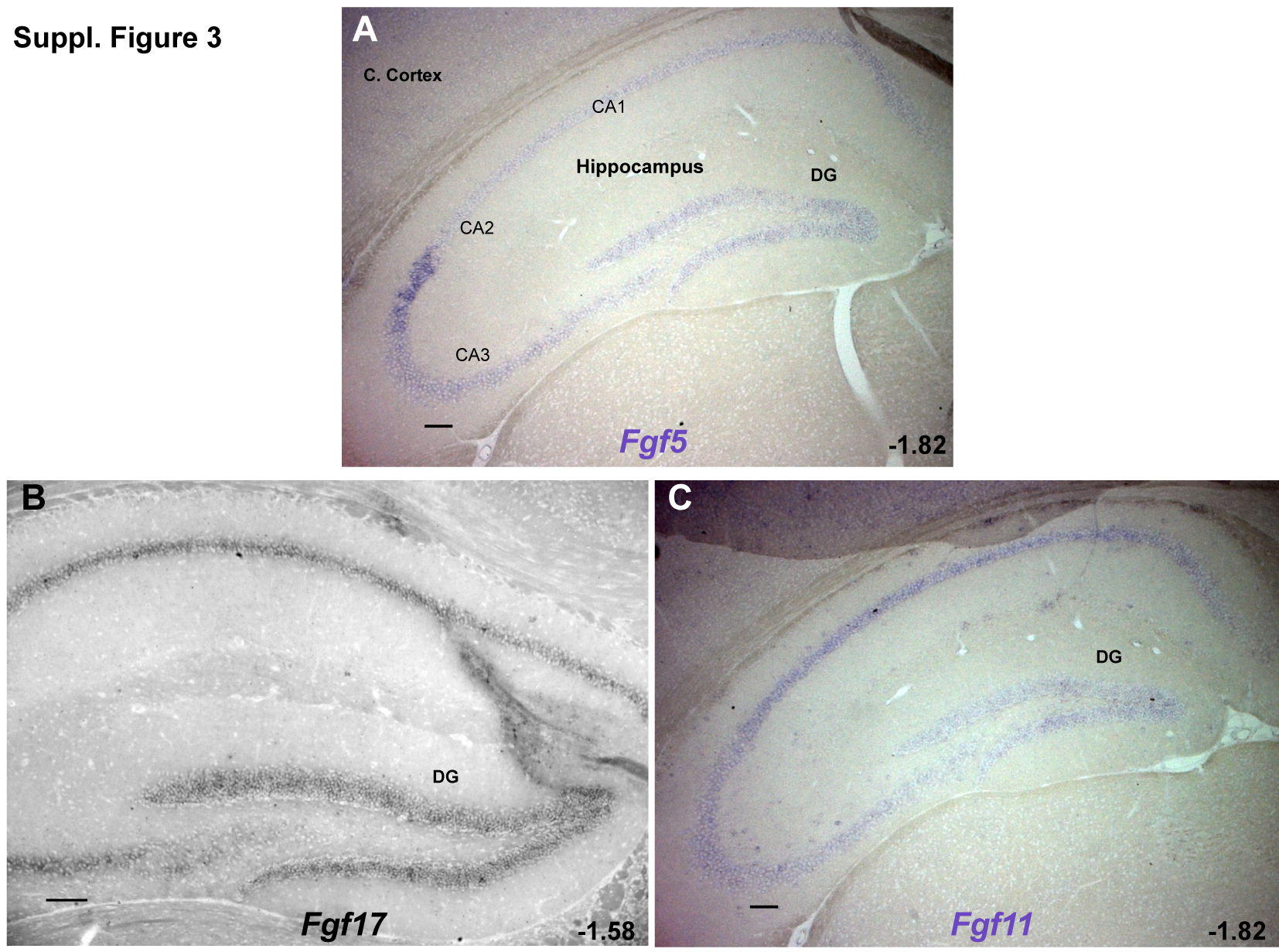

Supplement: Supplementary file 3 [file JNE-31-na-s003.tif]

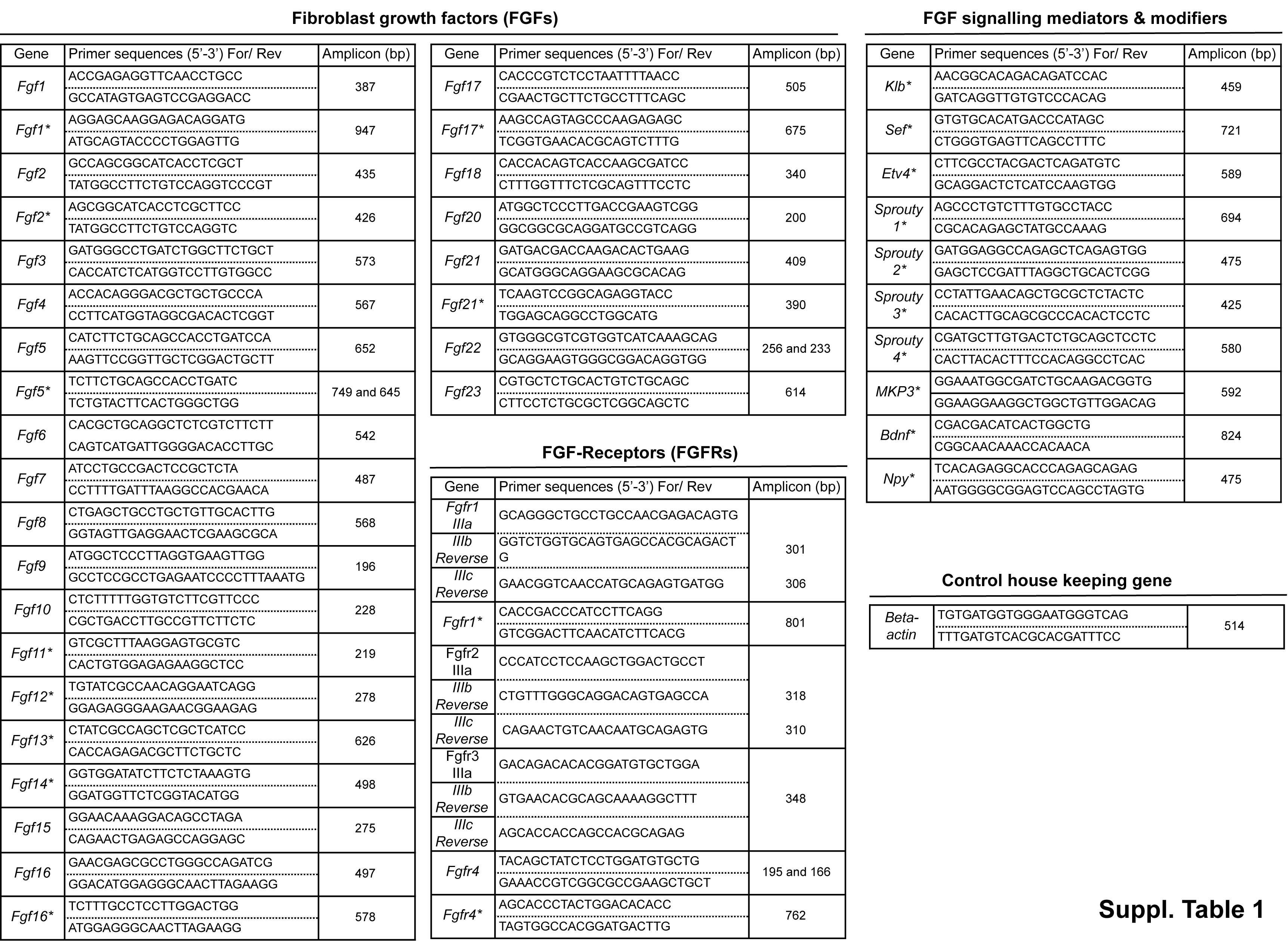

Supplement: Supplementary file 4 [file JNE-31-na-s004.tif]

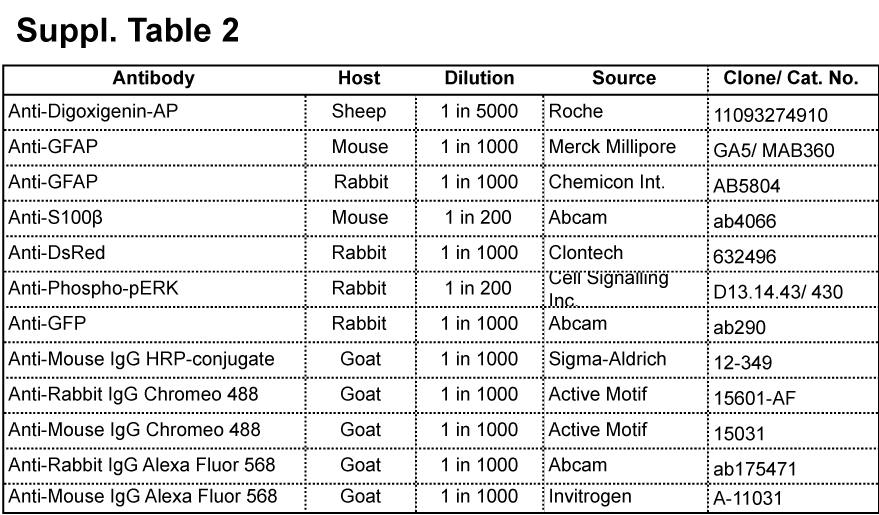

Supplement: Supplementary file 5 [file JNE-31-na-s005.tif]
